# Assessing Patient Radiation Exposure in Endoscopic Retrograde Cholangiopancreatography: A Multicenter Retrospective Analysis of Procedural Complexity and Clinical Factors

**DOI:** 10.3390/diagnostics14060656

**Published:** 2024-03-21

**Authors:** Touko Kaasalainen, Ekaterina Saukko, Outi Lindström, Marianne Udd, Sara Regnér, Arto Saarela, Ervin Toth, Gabriele Wurm Johansson, Anna-Leena Manninen, Juha Grönroos, Leena Kylänpää

**Affiliations:** 1 HUS Diagnostic Center, Radiology, University of Helsinki and Helsinki University Hospital, 00290 Helsinki, Finland; 2 Department of Radiology, Turku University Hospital and University of Turku, 20521 Turku, Finland; 3 HUS Abdominal Center, Endoscopy Department, University of Helsinki and Helsinki University Hospital, 00290 Helsinki, Finland; 4 Department of Clinical Sciences Malmö, Lund University, 22100 Malmö, Sweden; 5 Department of Surgery and Gastroenterology, Skåne University Hospital, 20502 Malmö, Sweden; 6 Department of Gastrointestinal Surgery, Oulu University Hospital, 90220 Oulu, Finland; 7 Department of Radiology, Oulu University Hospital, 90220 Oulu, Finland; 8 Division of Digestive Surgery and Urology, Turku University Hospital and University of Turku, 20521 Turku, Finland

**Keywords:** ERCP, fluoroscopy, radiation exposure, procedural complexity, PSC

## Abstract

Background and aims: Endoscopic retrograde cholangiopancreatography (ERCP) procedures can result in significant patient radiation exposure. This retrospective multicenter study aimed to assess the influence of procedural complexity and other clinical factors on radiation exposure in ERCP. Methods: Data on kerma-area product (KAP), air-kerma at the reference point (K_a,r_), fluoroscopy time, and the number of exposures, and relevant patient, procedure, and operator factors were collected from 2641 ERCP procedures performed at four university hospitals. The influence of procedural complexity, assessed using the American Society for Gastrointestinal Endoscopy (ASGE) and HOUSE complexity grading scales, on radiation exposure quantities was analyzed within each center. The procedures were categorized into two groups based on ERCP indications: primary sclerosing cholangitis (PSC) and other ERCPs. Results: Both the ASGE and HOUSE complexity grading scales had a significant impact on radiation exposure quantities. Remarkably, there was up to a 50-fold difference in dose quantities observed across the participating centers. For non-PSC ERCP procedures, the median KAP ranged from 0.9 to 64.4 Gy·cm^2^ among the centers. The individual endoscopist also had a substantial influence on radiation dose. Conclusions: Procedural complexity grading in ERCP significantly affects radiation exposure. Higher procedural complexity is typically associated with increased patient radiation dose. The ASGE complexity grading scale demonstrated greater sensitivity to changes in radiation exposure compared to the HOUSE grading scale. Additionally, significant variations in dose indices, fluoroscopy times, and number of exposures were observed across the participating centers.

## 1. Introduction

Endoscopic retrograde cholangiopancreatography (ERCP) is a minimally invasive procedure used for diagnostic and therapeutic purposes in the field of gastroenterology. It combines endoscopy and fluoroscopy to visualize and treat disorders of the bile ducts, pancreas, and gallbladder. In recent years, there has been a growing interest in improving and standardizing the quality of ERCP procedures. This drive stems from the shared goal of promoting best practices and evidence-based care for patients [1,2,3,4,5]. Practical guidelines on various aspects of quality indicators for ERCP have been proposed by esteemed organizations such as the American Society for Gastrointestinal Endoscopy (ASGE), American College of Gastroenterology (ACG), European Society for Gastrointestinal Endoscopy (ESGE), United European Gastroenterology (UEG), British Society of Gastroenterology (BSG), Canadian Association of Gastroenterology (CAG), and Saudi Gastroenterology Association (SGA) [6,7,8,9,10,11]. Apart from appropriate clinical indications, high cannulation and procedure success rates, and low adverse event rate, radiation exposure quantities have been recommended as quality indicators and performance measures for enhancing the quality of ERCP [7,8,9].

Radiation dose is a significant concern in ERCP due to the use of fluoroscopic equipment. Excessive radiation exposure poses potential health risks, including an increased change in radiation-induced malignancies. The risk of stochastic effects, such as cancer, is believed to increase linearly with radiation dose [12,13,14]. Therefore, optimizing radiation dose without compromising the diagnostic or therapeutic quality of the procedure is crucial. Modern fluoroscopy systems provide several radiation exposure quantities, including fluoroscopy time (FT), kerma-area product (KAP), cumulative air-kerma at the patient reference entrance point (K_a,r_), and the number of acquired radiographic images or frames taken during the procedure. Among these, KAP serves as a surrogate parameter for stochastic radiation effects (e.g., cancer), while K_a,r_ serves as a surrogate parameter for deterministic effects (e.g., tissue reactions).

The imaging technique used, such as fluoroscopy at different dose levels and pulse rates, acquisition of single radiographic images, or frame acquisition can impact patient radiation exposure. Additionally, factors such as the type of X-ray equipment, distance between the endoscopy and fluoroscopy monitors, anatomical location of the targeted pathology, pancreatic indication for the procedure, cannulation difficulty, experience of the endoscopist, annual volume, trainee involvement, procedural complexity, sphincterotomy, stent placement, balloon dilatation, stone extraction, and brushing have been identified as associated with increased radiation exposure in ERCP [15,16,17,18,19,20,21,22,23,24,25,26,27,28,29]. The degree of procedural complexity can significantly vary within the same ERCP procedure due to factors such as patient anatomy, clinical factors, pathology being treated, and disease severity [30]. As a result, the patient’s radiation exposure is predominantly influenced by procedural complexity and should be evaluated on an individual basis [30].

Currently, there are several grading scales available to quantify the degree of procedural complexity in ERCP. Schutz and Abbott [31] were the first to propose a 1 to 5 grading scale to objectively quantify the difficulty of ERCP procedures. They observed that technical success was dependent on the degree of difficulty, but complications were not. The ASGE working party later published a grading system for the complexity of major endoscopic procedures, including ERCPs, using scores ranging from 1 to 4. The scores were based on median ratings from experienced endoscopists [32]. The effectiveness of the ASGE grading system in predicting technical success and adverse events has not been fully validated in clinical practice [33]. Olsson et al. [34] developed a novel HOUSE grading scale for ERCP complexity, which classifies procedures into three categories aligned with modern endoscopic treatment procedures in ERCP. However, the aspect of radiation exposure was not considered during the development of these grading scales.

The objective of this retrospective study was to evaluate the impact of procedural complexity in ERCP on patient radiation exposure in a multicenter setting, utilizing the ASGE and HOUSE grading scales. Additionally, we aimed to identify the patient-, procedure-, and operator-related factors that influence radiation exposure quantities in patients undergoing ERCP.

## 2. Materials and Methods

### 2.1. Study Design and Data Collection

This retrospective multicenter study was carried out at four university hospitals, with three hospitals located in Finland and one in Sweden. Since this study was retrospective and noninterventional in nature, it did not require review and approval from an ethics committee in the Finnish centers. However, research permissions were acquired from each participating center, ensuring the appropriate use of secondary data for this study. Conversely, an ethical approval was obtained for the Swedish center by the Swedish Ethical Review Authority (Dnr 2020-03673). Informed patient consent was not deemed necessary due to this study’s retrospective design and noninterventional approach. The research was carried out in accordance with the Helsinki Declaration.

The ERCP procedures were performed between February 2016 and December 2019, with the data collection period varying across the centers. This period ranged from approximately one year to three years. A standardized ERCP data collection sheet was uniformly employed in all centers to gather information on patient demographics, procedural characteristics, and radiation exposure. Data were collected from various sources, including medical records, picture archiving and communication systems (PACS), and internal systems of the hospitals’ radiology and gastroenterology divisions. Patient demographics encompassed age, gender, height, weight, and body mass index (BMI). Procedural characteristics included whether the patient had a native papilla, a history of previous endoscopic sphincterotomy, the anesthetic technique used, the indication for ERCP, cannulation time, specific interventions performed, total procedure time, the endoscopist involved, and the operator responsible for the fluoroscopic equipment. The cannulation time was measured from the initial contact with the papilla to the successful cannulation of the desired duct. The complexity of the ERCP procedure was evaluated using the 4-point ASGE complexity grading scale [32] and 3-point HOUSE complexity grading scale [34]. For each procedure, radiation exposure quantities such as FT, KAP, K_a,r_, and the number of exposures were recorded. Additionally, device-specific information regarding the fluoroscopic equipment used in the ERCP procedures was collected. This information included the manufacturer and model of the device, the type of fluoroscopic equipment (stationary fluoroscopic system, multipurpose fluoroscopic c-arm system, and mobile c-arm), the year of installation, and the type and size of the image receptor.

The ERCP procedures performed for the diagnosis and follow-up of primary sclerosing cholangitis (PSC) were analyzed separately, as they typically involved a larger number of single image exposures compared to other ERCP procedures. The impact of ERCP procedural complexity level, as determined by the ASGE or HOUSE grading scales, and the endoscopist on radiation exposure quantities was then analyzed.

### 2.2. Statistical Analysis

The data collected from the four participating centers were combined and statistical analyses were accomplished without identifiable patient data. Preliminary analysis revealed significant differences in radiation exposure between the centers. Since the dose results from a single center (Center 2) had a dominant influence on the overall results, the analyses were performed using within-center analyses.

The data are presented as median (interquartile range [IQR], i.e., first quartile–third quartile). To compare categorical and continuous variables among patient characteristics, ERCP indications (non-PSC and PSC), centers, ERCP procedural complexity, and endoscopists, Fisher’s Exact test or Mann–Whitney U-test and Kruskal–Wallis test with a post hoc Bonferroni correction were employed, respectively. Spearman’s correlation coefficients were used to assess the strength of linear relationships between radiation exposure quantities and between BMI and KAP. All statistical tests were two-sided, and a *p*-value of <0.05 was considered statistically significant. The statistical analyses were performed using SPSS statistical software (IBM, Armonk, NY, USA, version 25.0).

## 3. Results

### 3.1. Clinical Features of Patients and Procedural Characteristics

A total of 2641 fluoroscopy-guided ERCP procedures were included in this study, with the distribution per center as follows: Center 1 (*n* = 1500), Center 2 (*n* = 696), Center 3 (*n* = 171), and Center 4 (*n* = 274). The patient and procedural characteristics are summarized in Table 1. Of these patients, 53% (*n* = 1397) were male. The age of the patients ranged from 2 to 100 years, with a BMI range of 10.2 kg/m^2^ to 68.1 kg/m^2^. Patients in the PSC ERCP group were significantly younger [41.0 (32.0–53.0) years vs. 68.0 (55.0–78.0) years, *p* < 0.001], taller [1.74 (1.65–1.80) m vs. 1.70 (1.63–1.78) m, *p* < 0.001], and had lower BMI [24.9 (22.6–27.2) kg/m^2^ vs. 25.5 (22.7–29.0) kg/m^2^, *p* = 0.003] compared to patients in the non-PSC ERCP group.

The primary indication for ERCP was common bile duct (CBD) stones (*n* = 1084, 36%) and the majority of the ERCP procedures (*n* = 1491, 57%) were performed on patients with native papilla. Nearly one-third of the patients (*n* = 774, 29%) had undergone previous biliary endoscopic sphincterotomy (EST), 6% (*n* = 147) had pancreatic EST, and 4% (*n* = 118) had both procedures. The most typical interventions during ERCP were biliary EST (*n* = 1387, 25%), CBD stone extraction (*n* = 1033, 19%), and biliary stent placement, exchange, or removal (*n* = 800, 14%). The majority of ERCP procedures were performed under the supervision of an anesthesiologist. The number of endoscopists performing ERCP in each center varied from 1 to 9, with a total of 20 endoscopists (gastrointestinal surgeons or gastroenterologists) conducting the procedures. The distribution of ERCP procedures performed by endoscopists based on ASGE and HOUSE complexity grading levels varied notably (Table 2). In two centers (Centers 1 and 2), the endoscopists had full control over the fluoroscopic equipment. In the other two centers (Centers 3 and 4), radiographers played a significant role in operating the fluoroscopic systems (e.g., collimating radiation fields, adjusting dose levels, varying projection angles, and moving equipment), while endoscopists were responsible for irradiating the patient. A total of six fluoroscopic systems from two manufacturers were used in the participating centers for ERCP procedures (Center 1: Siemens Artis Zee Multi-Purpose, Siemens Arcadis Avantic, and Siemens Cios Alpha (Siemens Healthineers, Erlangen, Germany); Center 2: Philips Allura Xper FD20 (Philips, Amsterdam, the Netherlands); Center 3: Siemens Artis Zee Multi-Purpose; and Center 4: Siemens Artis Zee Multi-Purpose). The fluoroscopic systems included multipurpose C-arm units and mobile C-arms, and all the devices were installed between the years 2009 and 2017. All the devices were utilizing flat-panel detectors except the Siemens Arcadis Avantic mobile C-arm at Center 1, which was an image intensifier system. All equipment used automatic exposure control.

### 3.2. Radiation Exposure Quantities of the Procedures

**Figure 1:**
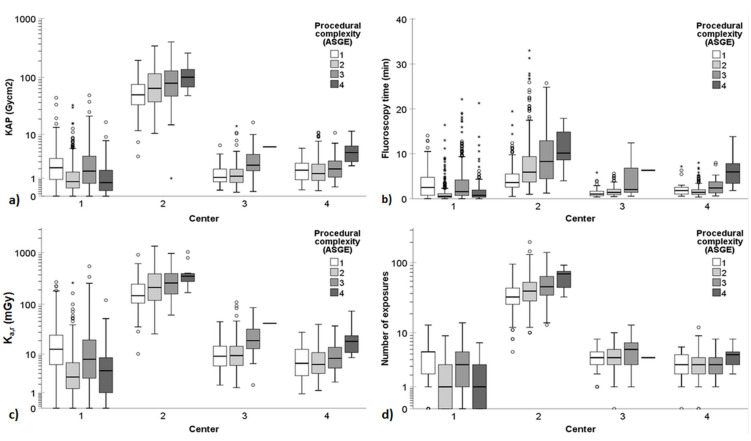
Patient radiation exposure quantities in all ERCP procedures (thus, containing data both from non-PSC and PSC procedures) according to the 4-point ASGE complexity grading scale. (**a**) KAP, (**b**) fluoroscopy time, (**c**) K_a,r_, and (**d**) number of exposures varied significantly between the participating centers. A logarithmic scale is used on the *y*-axis for KAP, K_a,r_, and number of exposures to better delineate the changes at low dose levels and low number of exposures. In the boxplots, circles and asterisks denote outliers and extreme cases, respectively. Outliers are defined as data points that fall beyond 3 standard deviations from the mean. Extreme cases are identified as data points lying outside of 1.5 times the interquartile range above the third quartile or below the first quartile in the boxplot.

**Figure 2:**
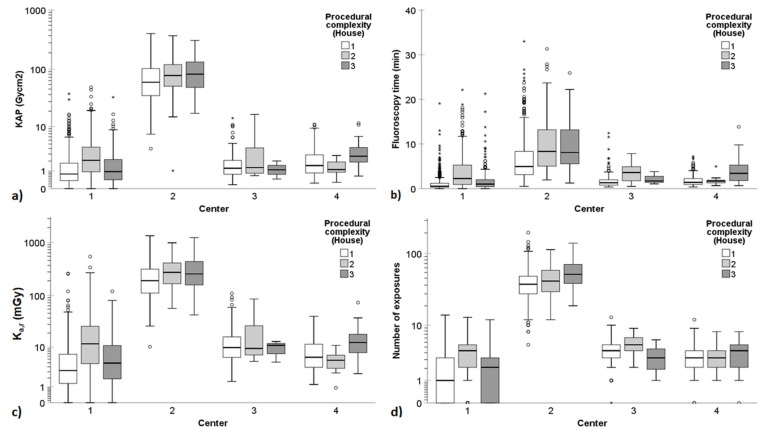
Patient radiation exposure quantities in all ERCP procedures (thus, containing data both from non-PSC and PSC procedures) according to the 3-point HOUSE complexity grading scale. (**a**) KAP, (**b**) fluoroscopy time, (**c**) K_a,r_, and (**d**) number of exposures varied significantly between the participating centers. A logarithmic scale is used on the *y*-axis for KAP, K_a,r_, and number of exposures to better delineate the changes at low dose levels and low number of exposures. In the boxplots, circles and asterisks denote outliers and extreme cases, respectively. Outliers are defined as data points that fall beyond 3 standard deviations from the mean. Extreme cases are identified as data points lying outside of 1.5 times the interquartile range above the third quartile or below the first quartile in the boxplot.

Table 3 and Figure 1 provide a summary of the radiation dose indices, fluoroscopy times, and number of exposures for the ERCP procedures conducted across the participating centers, categorized according to the ASGE complexity grading scale. Similarly, Table 4 and Figure 2 present the radiation exposure quantities in ERCPs across the participating centers based on the HOUSE scale. Figure 1 and Figure 2 encapsulate the radiation exposure quantities encompassing all ERCP procedures, whereas Table 3 and Table 4 show these quantities separately for non-PSC and PSC procedures. The findings revealed substantial variation in KAP, FT, K_a,r_, and number of exposures between the participating centers. Particularly, Center 2 exhibited significantly higher radiation exposure quantities compared to the other centers.

The accumulated KAP ranged from 0.01 to 400.93 Gy·cm^2^, FT varied from 1 s to 33 min, K_a,r_ spanned from 0.1 to 1335.4 mGy, and the number of exposures ranged from 0 to 192 images. The complexity level of the ERCP had a statistically significant impact on the radiation exposure quantities. Generally, as the complexity level increased, so did the radiation exposure quantity.

The ASGE complexity grading scale showed somewhat stronger correlation with radiation exposure quantities compared to the HOUSE grading scale. For instance, when examining the correlation between KAP and the grading scales in non-PSC procedures, the Spearman’s correlation coefficients for the ASGE grading scale ranged from 0.146 to 0.225 across the different centers, whereas the correlation coefficients for the HOUSE grading scale ranged from 0.022 to 0.174 (*p* < 0.05). PSC ERCPs, often classified as ASGE level 1 or 3, resulted in significantly higher KAP, K_a,r_, FT, and a greater number of single image acquisitions compared to non-PSC ERCPs at Center 1 (*p* < 0.001). However, no similar patterns were observed in other centers, possibly due to the small number of PSC ERCPs performed.

**Figure 3:**
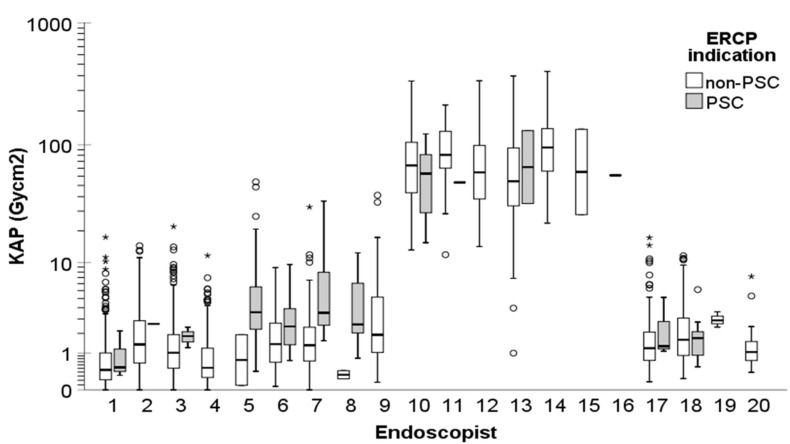
KAP of the non-PSC and PSC ERCP procedures across the endoscopists (the endoscopists #1–9 were from Center 1, #10–16 from Center 2, #17 from Center 3, and #18–20 from Center 4, respectively). A logarithmic scale is used on the *y*-axis to better delineate the changes at low dose levels. In the boxplots, circles and asterisks denote outliers and extreme cases, respectively. Outliers are defined as data points that fall beyond 3 standard deviations from the mean. Extreme cases are identified as data points lying outside of 1.5 times the interquartile range above the third quartile or below the first quartile in the boxplot.

The dose indices, FT, and number of exposures during ERCP procedures exhibited significant variations not only between the centers but also among the endoscopists (*p* < 0.001). Figure 3 provides a visualization of the KAP for non-PSC and PSC ERCP indications across different endoscopists. It was observed that endoscopists at Center 2 consistently utilized significantly higher doses, longer fluoroscopy times, and captured more images during the procedures compared to their counterparts in other centers (*p* < 0.001).

**Figure 4:**
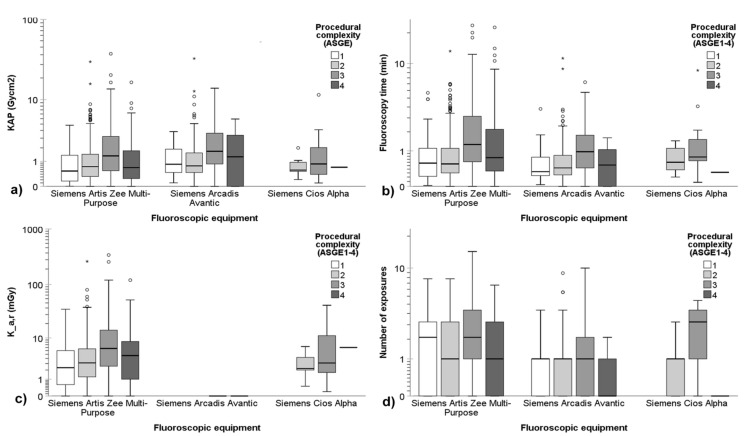
Device-specific comparative analysis of radiation exposure quantities in ERCP procedures for non-PSC indications for Center 1 equipment according to ASGE complexity grading levels: (**a**) KAP, (**b**) fluoroscopy time, (**c**) K_a,r_, and (**d**) number of exposures. A logarithmic scale is employed on the *y*-axis to enhance visualization of changes at low dose levels, shorter fluoroscopy times, and low number of exposures. In the boxplots, circles and asterisks denote outliers and extreme cases, respectively. Outliers are defined as data points that fall beyond 3 standard deviations from the mean. Extreme cases are identified as data points lying outside of 1.5 times the interquartile range above the third quartile or below the first quartile in the boxplot.

In Center 1, three distinct fluoroscopic systems were deployed for patient procedures. All ERCPs for PSC (*n* = 288) were conducted using a Siemens Artis Zee Multi-Purpose device, while all three systems were employed for non-PSC ERCPs: Siemens Artis Zee Multi-Purpose (*n* = 956), Siemens Arcadis Avantic (*n* = 233), and Siemens Cios Alpha (*n* = 23). Figure 4 illustrates a comparative analysis of radiation exposure quantities specific to each device for non-PSC ERCP procedures at Center 1, categorized based on ASGE complexity grading levels. Notably, no statistically significant differences were observed in KAP among the equipment (*p* = 0.548). However, FT (*p* < 0.001), K_a,r_ (*p* = 0.009), and number of exposures (*p* < 0.001) were significantly higher with the multipurpose C-arm system compared to the mobile C-arms. Nevertheless, the observed differences remained clinically insignificant.

As anticipated, patient BMI had an impact on patient radiation exposure. Higher BMI correlated with higher doses. The Spearman’s correlation coefficient between BMI and KAP exhibited varying degrees of association across the centers, ranging from weak to moderate (0.259 ≤ ρ ≤ 0.423). Furthermore, depending on the participating center, a moderate to very strong correlation was found between KAP and FT (0.530 ≤ ρ ≤ 0.827), a very strong correlation between KAP and K_a,r_ (0.929 ≤ ρ ≤ 0.983), and a moderate to strong correlation between KAP and number of exposures (0.470 ≤ ρ ≤ 0.685).

## 4. Discussion

This multicenter study aimed to investigate the impact of procedural complexity on patient radiation exposure in ERCP procedures, utilizing the ASGE and HOUSE grading scales. Additionally, this study examined the effects of patient BMI, ERCP indication (non-PSC vs. PSC), and endoscopists on radiation exposure quantities.

The findings of this study demonstrated that the complexity of ERCP procedures, as determined by the ASGE or HOUSE grading scales, significantly influenced dose indices, fluoroscopy times, and the number of exposures (Table 3 and Table 4 and Figure 1 and Figure 2). Generally, as the complexity level increased, so did the radiation exposure quantity. However, there were a few exceptions, particularly in the case of Center 1, which had quite a different distribution of procedures compared to other centers. The indications for ERCP and the interventions performed during the procedures varied among the centers. For instance, Center 1 conducted a majority of the PSC (*n* = 288/321, 90%) and double-balloon (*n* = 60/62, 97%) ERCP procedures. This primarily explains the discrepancies in dose results between Center 1 and the other centers. At Center 1, ERCP procedures performed for diagnosing and following up PSC, typically categorized as ASGE level 1 or 3 procedures, resulted in higher dose indices, longer fluoroscopy times, and a greater number of exposures compared to non-PSC ERCPs. No similar observations were made in the other centers. Furthermore, although double-balloon ERCPs do not require high radiation doses, they are still classified as ASGE level 4 due to their complex nature. Therefore, the median doses of ASGE level 4 procedures were lower than those of level 3 procedures in Center 1. Although this study revealed statistically significant disparities in radiation exposure quantities across the ERCP complexity grading levels, these variations generally held limited clinical significance, except for the case of Center 2.

Overall, significant variation in KAP, FT, K_a,r_, and the number of exposures were observed among the participating centers. Particularly, Center 2 exhibited KAP values up to 50 times higher, FT up to 5 times longer, K_a,r_ up to 35 times higher, and the number of exposures up to 20 times greater compared to the other centers. These differences can largely be attributed to potential disparities in dose optimization and radiation safety practices, as well as the used fluoroscopic equipment. As a direct outcome of this study and heightened awareness regarding elevated radiation doses, Center 2 has recently undergone changes in equipment and instituted an educational program. Moreover, there were notable variations in working practices among the participating centers. In Centers 1 and 2, endoscopists held full control over the fluoroscopic equipment. Conversely, in Centers 3 and 4, radiographers were responsible for collimating radiation fields, and upon endoscopists’ requests, they adjusted equipment positioning, altered dose levels, manipulated projection angles, and adapted detector-to-patient distances. Also in these centers, the endoscopists were responsible for irradiating the patient using foot pedals. These discrepancies in working practices may, at some point, contribute to the observed variations in radiation doses, alongside potential differences in the employed imaging protocols and the nature of procedures performed. It is noteworthy to mention that the fluoroscopic systems were uniform in Centers 1, 3, and 4.

When the ASGE and HOUSE grading scales for procedural complexity in ERCP procedures were developed, the aspect of radiation exposure was not taken into consideration. However, the findings of this study suggest that the ASGE scale correlates better with the radiation exposure quantities compared to the HOUSE grading scale. Previous studies have also reported the impact of ASGE grading on radiation dose. For instance, Saukko et al. [24] observed significantly higher doses and fluoroscopy times for procedures categorized as complexity level 3 compared to level 1 and level 2 procedures. The median KAP for ERCPs in their study was 1.83 Gy·cm^2^ (IQR: 1.20–2.90 Gy·cm^2^). Similarly, Kaasalainen et al. [29] reported that the ASGE level impacted radiation exposure. They also determined higher doses for ERCPs performed for PSC indication compared to other indications. The median KAP per ERCP in their study was 1.0 Gy·cm^2^ (0.8 and 1.3 Gy·cm^2^ for non-PSC and PSC ERCP, respectively), with the third quartile being 2.3 Gy·cm^2^. O’Connor et al. [35] reported higher KAP values than the previous studies. They found mean KAP per procedure ranging from 5.4 to 14.5 Gy·cm^2^, with third quartiles ranging from 7.9 to 19.6 Gy·cm^2^, depending on the endoscopy site and the image intensifier fluoroscopy system used. Nevertheless, these doses were still much lower than the doses reported in this study for Center 2 procedures, highlighting the need for optimization of imaging protocols and working practices at Center 2.

Significant variations in dose quantities were observed among the 20 endoscopists (Figure 3). As expected, the highest doses were recorded among endoscopists at Center 2. Additionally, at Center 1, gastroenterologists who primarily performed ERCPs for diagnosing and following up PSC patients, as well as performing dilatations and stent placements for these patients, exhibited significantly higher radiation exposure quantities compared to gastrointestinal surgeons who performed most of the other ERCP procedures. These differences could be attributed to factors such as experience, variations in radiation safety practices, and the unique skills of individual endoscopists leading to the accumulation of certain procedures under specific physicians.

The clinical impact of radiation exposure is a multifaceted issue. Typically, higher radiation doses yield superior image quality with reduced image noise, potentially aiding gastroenterologists and gastrointestinal surgeons in decision making during ERCP procedures. However, it is imperative to acknowledge the inherent health risks associated with ionizing radiation. Skin doses resulting from ERCP procedures were found to be below the threshold levels for deterministic harmful effects of ionizing radiation. In our study, the highest cumulative air-kerma at the patient reference entrance point was approximately 1.3 Gy, well below the 2 Gy threshold for temporary skin damage (transient erythema). In ERCP, the adverse effects of ionizing radiation are related to an increased risk of radiation-induced cancer. The linear-no-threshold model suggests that the likelihood of radiation-induced cancer increases linearly with exposure. Adhering to the ALARA principle, it is essential to minimize radiation exposure to the lowest feasible levels while ensuring adequate image quality for accurate diagnostics and safe image-guided interventions. In our study, the median KAP for non-PSC ERCP procedures was 0.9 Gy·cm^2^ in Center 1, compared to 64.4 Gy·cm^2^ in Center 2. These KAP values correspond to effective doses of 0.2 mSv and 16.7 mSv, respectively, utilizing the conversion coefficient provided in the NCRP 160 report [36] for ERCP procedures (0.26 mSv/(Gy·cm^2^)). The highest estimated effective dose from a single ERCP procedure in this study was approximately 100 mSv. However, it is noteworthy to mention that KAP-to-effective-dose conversion coefficients depend on various factors such as X-ray tube voltage, total filtration, patients’ gender, and age. Thus, the estimated effective dose should be interpreted with caution. The worldwide average natural radiation exposure to humans is approximately 2.4 mSv per year [37]. Hence, a single ERCP procedure in our study resulted in an effective dose equivalent to one month to seven years of natural background radiation, on average. The standard mortality risk rate attributed to ionizing radiation is estimated to be 5% per sievert. Therefore, the additional cancer risk from a single ERCP procedure for a single patient is minimal to low. However, it is crucial to emphasize that effective dose calculations and related mortality risk rates from a single exposure or procedure should not be applied on an individual basis. The effective dose can be utilized in medicine as a component of decision-making processes and justification of procedures, selection of imaging techniques, optimization of imaging protocols, reporting of unintended exposures, calculations of collective effective dose, and communication with health professionals and patients [38,39].

This study has several limitations that should be acknowledged. Firstly, the participation was limited to only four different centers from two countries. Additionally, the number of fluoroscopic equipment and endoscopists involved was relatively small, with six systems and twenty endoscopists. Secondly, there was variability in the distribution of ERCP indications and interventions among the centers and endoscopists. Some endoscopists performed only a few fluoroscopic procedures, which may introduce some limitations in the statistical analysis. Although the ASGE and HOUSE procedural complexity grading scales were utilized, comparing radiation exposure quantities across centers and endoscopists may still pose challenges. This study categorized non-PSC and PSC procedures into separate groups, with most PSC procedures and double-balloon interventions being performed in a single center. Furthermore, the evaluation of ERCP complexity level involved multiple endoscopists, which could have introduced some variability in the grading used, irrespective of the guidelines. Additionally, the classification of procedures into two groups (non-PSC vs. PSC) may not be ideal, as PSC ERCP procedures, for example, can exhibit significant variation. Some publications suggest evaluating diagnostic and therapeutic ERCPs separately. However, the classification into non-PSC and PSC ERCPs in our study was chosen to further categorize procedures in a way that is relevant in terms of radiation exposure. One notable difference is that in PSC cases, exposures are necessary to monitor changes in strictures during follow-up, potentially indicating the development of malignancy. The diagnosis of PSC can often be established through magnetic resonance imaging. However, brush cytology of strictures is usually needed. We believe that separately displaying the PSC group is important, given that PSC patients tend to be younger and may require repeated ERCP procedures. ERCP for PSC was classified as ASGE 1 in 64% of cases, indicating that it was primarily diagnostic, consisting of visualization with or without brush cytology. Lastly, the data regarding the procedure, ERCP indications, endoscopists’ experience, and patients’ characteristics may not have been homogenous enough to draw definite conclusions about the risks of radiation exposure in ERCP as a procedure itself. For example, some degree of variation in experience is inevitable among 20 endoscopists, although all were considered experienced. Upon analyzing the results according to each endoscopist (as shown in Table 2), we observed the highest radiation exposure among the most experienced ERCP endoscopists. This may be attributed to their involvement in more challenging cases. Achieving homogenous data in this type of clinical study is extremely challenging. Even for seemingly homogenous indications such as biliary stones, biliary strictures, or chronic pancreatitis, there can be considerable differences in difficulty levels. Additionally, variations in fluoroscopy usage and protocols (as well as traditions or habits) further contribute to discrepancies in radiation exposure. Practices regarding the use of fluoroscopy vary widely among centers, leading to differing radiation levels despite similar clinical outcomes. There are no unanimous practices regarding which types of images should be saved and how to use fluoroscopy effectively. For instance, some ERCP endoscopists routinely verify scope position before cannulation, while others may employ fluoroscopy only after successful guidewire insertion. Some centers even use fluoroscopy for preintubation of the duodenoscope to rule out perforation in uncertain cases. Therefore, there is a clear need for more specific recommendations on the appropriate use of fluoroscopy to minimize radiation exposure. To address the issue of data homogeneity, conducting a prospective study could lead to more homogeneity to a certain extent and facilitate the derivation of more conclusive findings. Nevertheless, our research findings depict a real-life scenario within a multicenter environment, where multiple endoscopists perform various types of ERCP procedures using different fluoroscopic systems.

## 5. Conclusions

In conclusion, this study revealed that procedural complexity plays a significant role in determining radiation exposure quantities in ERCP procedures. The ASGE complexity grading scale demonstrated a stronger correlation with radiation exposure quantities compared to the HOUSE grading scale. There was a notable variation in KAP, FT, K_a,r_, and the number of exposures among the participating centers, with one center exhibiting patient exposures up to 50 times higher than the other centers. Furthermore, patient size, the indication for ERCP, interventions performed during the procedure, and the expertise of the endoscopists were identified as significant factors impacting dose indices, fluoroscopy times, and the number of exposures. These findings emphasize the importance of optimizing the used imaging protocols and improving working practices to minimize radiation exposure during ERCP procedures.

## Figures and Tables

**Table 1:** Patient and procedural characteristics of 2641 patients who underwent ERCP.

Variables	Total
Gender (*n* = 2640) [*n* (%)]	
Female	1243 (47.1)
Male	1397 (52.9)
Age (y, *n =* 2640) [median (IQR)]	66.0 (52.0–76.7)
BMI (kg/m^2^, *n* = 2512) [median (IQR)]	25.4 (22.6–28.8)
Native papilla (*n* = 2640) [*n* (%)]	
Yes	1491 (56.5)
No	1133 (42.9)
Other (e.g., hepaticojejunostomy)	16 (0.6)
Previous EST (*n* = 2630) [*n* (%)]	
No	1591 (60.5)
Biliary EST only	774 (29.4)
Pancreatic EST only	147 (5.6)
Biliary and pancreatic EST	118 (4.5)
Anesthetic technique (*n* = 2632) [*n* (%)]	
Endoscopist-directed	714 (27.1)
Anesthesiologist-directed	1918 (72.9)
Indication (*n* = 3026) [*n* (%)]	
CBD stone	1084 (35.8)
Biliary stricture	818 (27.0)
PSC	321 (10.6)
Post LCC	116 (3.8)
Post LTx	84 (2.8)
Chronic pancreatitis	235 (7.8)
Acute pancreatitis	89 (2.9)
Pseudocyst	126 (4.2)
Other	153 (5.1)
Total cannulation time (min, *n* = 756) [median (IQR)]	1.08 (0.25–5.67)
Types of interventions (*n* = 5519) [*n* (%)]	
Biliary EST	1387 (25.1)
CBD stone extraction	1033 (18.7)
Biliary plastic stent placement, exchange, or removal	800 (14.5)
ERC cytology	691 (12.5)
Biliary dilatation	407 (7.4)
Metal stent placement	359 (6.5)
Pancreatic stent placement, exchange, or removal	259 (4.7)
Pancreatic EST	190 (3.4)
Pancreatic dilatation	158 (2.9)
ERP cytology	62 (1.1)
Pseudocystogastrostomy	26 (0.5)
Prophylactic pancreatic stent	29 (0.6)
Spyglass	56 (1.0)
Double-balloon ERCP	62 (1.1)
Total procedural time (min, *n* = 2555) [median (IQR)]	22.0 (15.0–33.0)
Operator of fluoroscopy equipment (*n* = 2640) [*n* (%)]	
Endoscopist	2195 (83.1)
Radiographer	445 (16.9)

ERCP, endoscopic retrograde cholangiopancreatography; BMI, body mass index; EST, endoscopic sphincterotomy; CBD, common bile duct; PSC, primary sclerosing cholangitis; LCC, laparoscopic cholecystectomy; LTx, liver transplantation; IQR, interquartile range; ERC, endoscopic retrograde cholangiography; and ERP, endoscopic retrograde pancreatography.

**Table 2:** Distribution of ERCP procedures performed by endoscopists based on ASGE and HOUSE complexity grading levels.

Endoscopist	Non-PSC/PSC	ASGE Complexity (Non-PSC/PSC)				HOUSE Complexity (Non-PSC/PSC)		
		Level 1	Level 2	Level 3	Level 4	Level 1	Level 2	Level 3
Center 1								
#1	209/4	9/0	69/0	72/2	59/2	82/0	53/1	74/3
#2	230/1	17/1	98/0	95/0	20/0	108/0	68/1	54/0
#3	331/3	48/0	118/0	148/1	17/2	169/0	114/1	48/2
#4	252/0	15/0	170/0	65/0	2/0	180/0	42/0	30/0
#5	2/178	1/117	0/9	0/52	1/0	0/1	1/176	1/1
#6	25/54	1/35	5/4	2/15	17/0	6/0	2/54	17/0
#7	94/19	6/17	63/0	24/2	1/0	71/0	15/19	8/0
#8	2/29	0/18	0/1	0/10	2/0	1/0	0/29	1/0
#9	67/0	0/0	23/0	38/0	6/0	22/0	27/0	18/0
Center2								
#10	191/6	33/1	129/2	23/3	6/0	145/0	18/6	28/0
#11	75/1	22/0	47/0	5/0	1/1	64/0	2/0	9/1
#12	172/0	47/0	110/0	15/0	0/0	148/0	4/0	20/0
#13	184/2	23/0	133/2	25/0	3/0	127/0	44/2	13/0
#14	62/0	14/0	34/0	13/0	1/0	56/0	4/0	2/0
#15	2/0	0/0	2/0	0/0	0/0	2/0	0/0	0/0
#16	1/0	0/0	0/0	1/0	0/0	1/0	0/0	0/0
Center 3								
#17	167/4	40/3	107/1	19/0	1/0	156/1	9/2	2/1
Center 4								
#18	236/20	17/13	164/5	45/2	10/0	191/0	6/16	39/4
#19	3/0	1/0	2/0	0/0	0/0	3/0	0/0	0/0
#20	15/0	0/0	14/0	1/0	0/0	13/0	2/0	0/0

ASGE, the American Society for Gastrointestinal Endoscopy and PSC, primary sclerosing cholangitis.

**Table 3:** Dose indices and fluoroscopy times of the ERCP procedures performed at different centers for non-PSC and PSC indications according to ASGE procedural complexity level grading.

Center and Complexity Level (ERCPs Without PSC/with PSC)	Fluoroscopy Time, min		KAP_,_ Gy·cm^2^		K_a,r,_ mGy		Number of Exposures	
	w/o PSC	PSC	w/o PSC	PSC	w/o PSC	PSC	w/o PSC	PSC
Center 1 (1212/288)	0.7 (0.4–1.6)	4.8 (2.7–7.6)	0.9 (0.4–1.9)	3.0 (2.0–5.5)	3.7 (1.6–9.1)	19.9 (12.5–39.0)	2 (0–3)	5 (5–6)
Level 1 (97/188)	0.5 (0.2–1.1)	4.1 (2.3–6.1)	0.6 (0.2–1.4)	2.6 (1.8–4.7)	2.2 (0.6–5.5)	16.8 (10.6–32.3)	1 (0–3)	5 (4–6)
Level 2 (546/14)	0.5 (0.3–1.0)	7.0 (4.4–8.7)	0.7 (0.4–1.5)	4.7 (2.5–5.8)	2.9 (1.2–6.0)	32.3 (22.6–53.8)	1 (0–2)	6 (5–6)
Level 3 (444/82)	1.2 (0.6–2.9)	7.5 (5.2–9.8)	1.3 (0.6–3.0)	4.0 (2.4–6.7)	5.9 (2.4–14.2)	31.5 (17.3–53.3)	2 (1–4)	5 (5–6)
Level 4 (125/4)	0.7 (0.3–1.9)	1.3 (1.0–2.7)	0.7 (0.2–1.7)	1.1 (0.4–2.0)	4.3 (1.0–8.4)	6.0 (3.9–8.3)	1 (0–3)	4 (3–4)
*p*-value (complexity levels)	<0.001	<0.001	<0.001	<0.001	<0.001	<0.001	<0.001	0.002
*p*-value (PSC vs. w/o PSC)	<0.001		<0.001		<0.001		<0.001	
Center 2 (687/9)	5.5 (3.5–9.4)	4.5 (3.9–6.9)	64.4 (38.1–106.7)	49.6 (32.5–83.0)	207.1 (120.1–350.7)	264.3 (118.7–461.4)	40 (29–52)	38 (36–59)
Level 1 (139/1)	3.6 (2.6–5.5)	3.1 (3.1–3.1)	50.3 (33.7–76.2)	49.6 (49.6–49.6)	146.1 (104.6–246.8)	243.4 (243.4–243.4)	33 (26–44)	38 (38–38)
Level 2 (455/4)	5.9 (3.7–9.4)	6.5 (6.5–18.9)	64.8 (38.0–114.7)	103.1 (57.8–127.1)	207.3 (117.8–381.8)	510.1 (290.1–580.7)	40 (29–52)	62 (48–76)
Level 3 (82/3)	8.3 (4.9–13.9)	2.0 (2.0–4.5)	81.6 (48.3–132.4)	27.1 (15.0–67.2)	259.5 (161.5–439.3)	113.0 (61.5–273.1)	47 (35–65)	35 (23–36)
Level 4 (11/1)	10.3 (9.1–16.2)	4.0 (4.0–4.0)	108.1 (68.7–143.6)	48.7 (48.7–48.7)	363.3 (289.3–400.8)	264.3 (264.3–264.3)	70 (47–78)	43 (43–43)
*p*-value (complexity levels)	<0.001	0.099	<0.001	0.338	<0.001	0.338	<0.001	0.118
*p*-value (PSC vs. w/o PSC	0.771		0.430		0.456		0.532	
Center 3 (167/4)	1.4 (0.8–2.3)	2.9 (2.0–4.0)	1.2 (0.7–2.0)	1.3 (1.2–2.2)	9.7 (5.7–16.0)	11.0 (10.3–14.3)	4 (3–6)	5 (4–6)
Level 1 (40/3)	0.9 (0.7–1.5)	2.1 (1.7–3.8)	1.0 (0.7–1.9)	1.3 (1.1–1.3)	8.9 (5.3–15.2)	10.8 (8.6–11.1)	4 (3–5)	5 (1–5)
Level 2 (107/1)	1.4 (0.9–2.0)	4.7 (4.7–4.7)	1.2 (0.7–1.8)	4.7 (4.7–4.7)	9.3 (5.6–14.4)	23.9 (23.9–23.9)	4 (3–5)	8 (8–8)
Level 3 (19/0)	2.0 (1.4–6.8)	–	2.3 (1.7–4.1)	–	19.1 (13.0–32.3)	–	6 (3–7)	–
Level 4 (1/0)	6.3 (6.3–6.3)	–	5.8 (5.8–5.8)	–	42.2 (42.2–42.2)	–	4 (4–4)	–
*p*-value (complexity levels)	0.002	0.500	0.003	0.500	0.003	0.500	0.320	0.500
*p*-value (PSC vs. w/o PSC	0.036		0.408		0.447		0.622	
Center 4 (254/20)	1.6 (1.0–2.6)	1.8 (1.5–2.2)	1.5 (0.9–2.8)	1.7 (0.9–2.0)	7.0 (3.9–12.5)	5.6 (3.9–7.4)	3 (2–4)	3 (2–4)
Level 1 (18/13)	2.4 (1.0–2.8)	1.7 (1.4–2.1)	2.2 (0.9–3.5)	1.6 (0.9–1.8)	10.6 (2.9–15.4)	5.4 (3.4–6.3)	3 (2–5)	3 (3–4)
Level 2 (180/5)	1.4 (1.0–2.0)	1.7 (1.7–1.9)	1.4 (0.8–2.6)	1.6 (0.9–1.9)	5.9 (3.6–11.1)	6.0 (4.4–8.4)	3 (2–4)	2 (2–3)
Level 3 (46/2)	2.2 (1.3–3.8)	3.1 (2.8–3.4)	1.8 (1.1–2.9)	3.8 (1.9–5.6)	8.0 (4.9–13.7)	16.7 (8.1–25.3)	3 (2–4)	5 (4–5)
Level 4 (10/0)	6.0 (3.4–7.8)	–	4.5 (2.8–6.0)	–	18.4 (10.8–23.9)	–	5 (3–5)	–
*p*-value (complexity levels)	<0.001	0.123	<0.001	0.252	<0.001	0.116	0.056	0.080
*p*-value (PSC vs. w/o PSC	0.567		0.521		0.224		0.158	
*p*-value (centers)	<0.001	<0.001	<0.001	<0.001	<0.001	<0.001	<0.001	<0.001

ERCP, endoscopic retrograde cholangiopancreatography; KAP, kerma-area product; K_a,r_, air-kerma at reference point; and PSC: primary sclerosing cholangitis. Results are given as median (IQR).

**Table 4:** Dose indices and fluoroscopy times of the ERCP procedures performed at different centers for non-PSC and PSC indications according to HOUSE procedural complexity level grading.

Center and Complexity Level (ERCPs Without PSC/with PSC)	Fluoroscopy Time, min		KAP_,_ Gy·cm^2^		K_a,r,_ mGy		Number of Exposures	
	w/o PSC	PSC	w/o PSC	PSC	w/o PSC	PSC	w/o PSC	PSC
Center 1 (1212/288)	0.7 (0.4–1.6)	4.8 (2.7–7.6)	0.9 (0.4–1.9)	3.0 (2.0–5.5)	3.7 (1.6–9.1)	19.9 (12.5–39.0)	2 (0–3)	5 (5–6)
Level 1 (639/1)	0.6 (0.3–1.2)	4.2 (4.2–4.2)	0.8 (0.4–1.7)	3.3 (3.3–3.3)	3.0 (1.3–6.9)	20.5 (20.5–20.5)	1 (0–3)	5 (5–5)
Level 2 (322/281)	1.1 (0.5–2.1)	4.8 (2.9–7.6)	1.1 (0.5–2.2)	3.0 (2.0–5.6)	4.8 (2.1–10.5)	20.5 (13.0–39.4)	2 (1–3)	5 (5–6)
Level 3 (251/6)	1.0 (0.4–2.0)	1.3 (0.8–4.2)	0.9 (0.4–2.2)	1.1 (0.5–1.9)	4.4 (1.8–10.7)	6.0 (3.0–9.7)	2 (0–3)	4 (2–4)
*p*-value (complexity levels)	<0.001	0.085	0.003	0.006	<0.001	0.003	<0.001	0.049
*p*-value (PSC vs. w/o PSC)	<0.001		<0.001		<0.001		<0.001	
Center 2 (687/9)	5.5 (3.5–9.4)	4.5 (3.9–6.9)	64.4 (38.1–106.7)	49.6 (32.5–83.0)	207.1 (120.1–350.7)	264.3 (118.7–461.4)	40 (29–52)	38 (36–59)
Level 1 (543/0)	5.0 (3.1–8.3)	–	60.1 (35.5–102.9)	–	190.8 (111.2–315.8)	–	38 (28–49)	–
Level 2 (72/8)	9.0 (5.2–13.3)	5.3 (3.5–8.6)	79.0 (55.5–120.2)	58.4 (29.8–103.1)	277.3 (168.4–400.8)	258.3 (115.9–510.1)	42 (29–59)	38 (36–62)
Level 3 (72/1)	8.2 (5.7–13.2)	4.0 (4.0–4.0)	83.4 (49.4–134.5)	48.7 (48.7–48.7)	254.8 (157.6–449.0)	264.3 (264.3–264.3)	52 (39–71)	43 (43–43)
*p*-value (complexity levels)	<0.001	0.889	<0.001	0.889	<0.001	1.000	<0.001	1.000
*p*-value (PSC vs. w/o PSC	0.771		0.430		0.456		0.532	
Center 3 (167/4)	1.4 (0.8–2.3)	2.9 (2.0–4.0)	1.2 (0.7–2.0)	1.3 (1.2–2.2)	9.7 (5.7–16.0)	11.0 (10.3–14.3)	4 (3–6)	5 (4–6)
Level 1 (156/1)	1.3 (0.8–2.0)	1.7 (1.7–1.7)	1.2 (0.7–2.0)	1.3 (1.3–1.3)	9.7 (5.9–16.0)	11.1 (11.1–11.1)	4 (3–5)	5 (5–5)
Level 2 (9/2)	3.6 (1.5–5.1)	3.4 (2.1–4.7)	1.0 (0.8–3.1)	3.0 (1.3–4.7)	9.3 (5.5–29.0)	16.3 (8.6–23.9)	4 (4–6)	7 (5–8)
Level 3 (2/1)	1.4 (1.1–1.7)	3.8 (3.8–3.8)	1.2 (0.5–1.9)	1.1 (1.1–1.1)	8.8 (4.7–12.9)	10.8 (10.8–10.8)	5 (3–6)	1 (1–1)
*p*-value (complexity levels)	0.070	0.407	0.811	0.407	0.730	0.861	0.721	0.325
*p*-value (PSC vs. w/o PSC	0.036		0.408		0.447		0.622	
Center 4 (254/20)	1.6 (1.0–2.6)	1.8 (1.5–2.2)	1.5 (0.9–2.8)	1.7 (0.9–2.0)	7.0 (3.9–12.5)	5.6 (3.9–7.4)	3 (2–4)	3 (2–4)
Level 1 (207/0)	1.4 (1.0–2.3)	–	1.4 (0.8–2.7)	–	6.1 (3.6–11.4)	–	3 (2–4)	–
Level 2 (8/16)	1.7 (1.3–1.8)	1.7 (1.2–2.0)	1.0 (0.9–1.5)	1.6 (0.9–1.8)	4.8 (3.5–7.0)	5.5 (3.3–6.7)	3 (2–5)	3 (3–4)
Level 3 (39/4)	4.0 (1.8–5.5)	2.4 (1.8–3.1)	2.6 (1.7–3.9)	2.0 (1.7–3.9)	12.7 (8.4–18.0)	7.7 (6.3–16.7)	4 (2–5)	4 (3–5)
*p*-value (complexity levels)	<0.001	0.178	<0.001	0.122	<0.001	0.122	0.009	0.682
*p*-value (PSC vs. w/o PSC	0.567		0.521		0.224		0.158	
*p*-value (centers)	<0.001	<0.001	<0.001	<0.001	<0.001	<0.001	<0.001	<0.001

ERCP, endoscopic retrograde cholangiopancreatography; KAP, kerma-area product; K_a,r_, air-kerma at reference point; and PSC: primary sclerosing cholangitis. Results are given as median (IQR).

## Data Availability

Individual-level data cannot be shared openly due to restrictions imposed by the research permit.
